# Supplementary figures and images for: Dex-Benchmark: datasets and code to evaluate algorithms for transcriptomics data analysis
Source: PeerJ. 2023 Nov 8;11:e16351. doi: 10.7717/peerj.16351 (PMC10638921; doi:10.7717/peerj.16351)

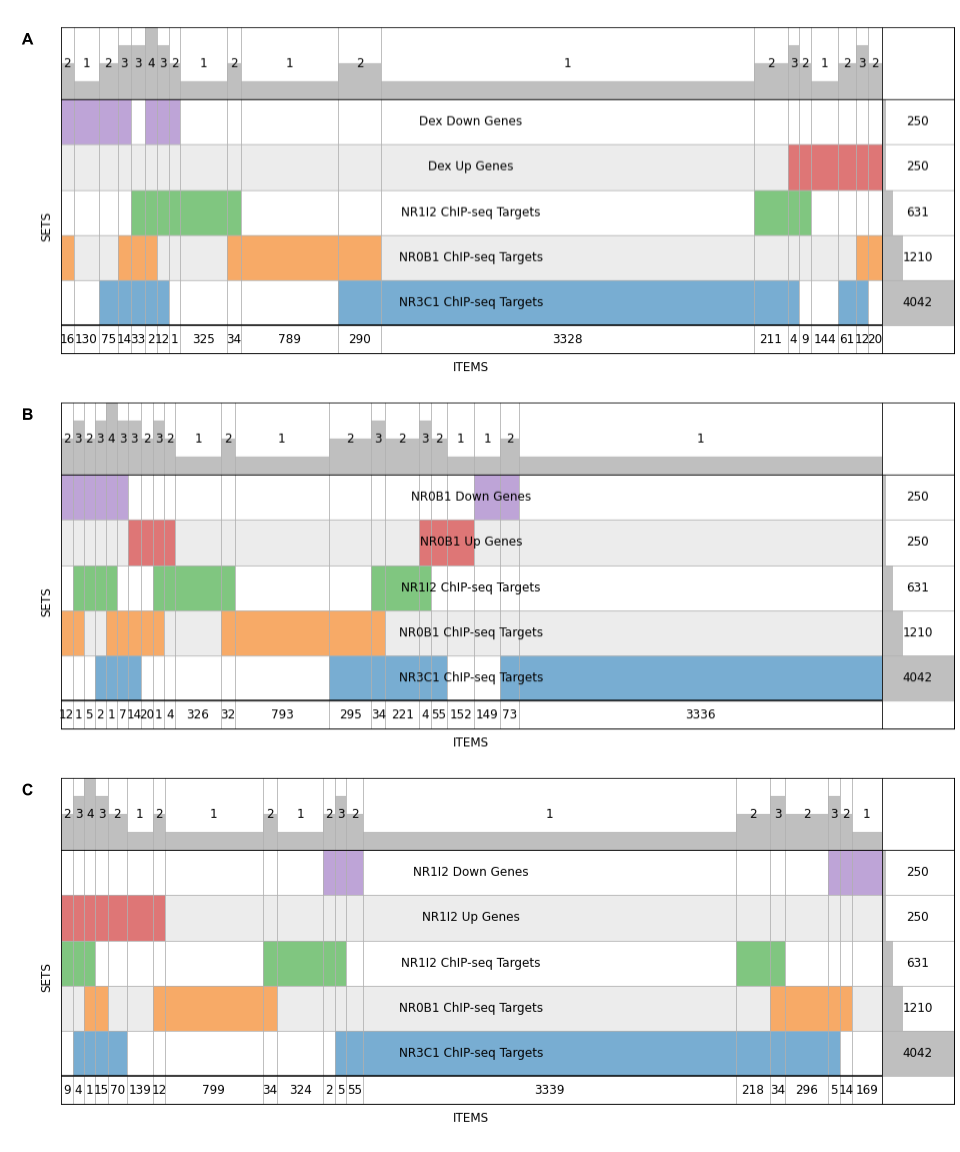

Supplement: Supplemental Information 1 — (A) Overlap between dexamethasone up/down gene sets and NR3C1, NR0B1, NR1I2 target gene sets. Each row indicates a different set. The column labels at the top display the number of overlapping sets, while the column labels at the bottom display the number of overlapping items. (B) Supervenn diagram showing overlap between NR0B1 CRISPR KO consensus up/down genes and all three target gene sets. Column labels are the same as in (A). (C) Supervenn diagram showing overlap between NR1I2 CRISPR KO consensus up/down genes and all three target gene sets. Column labels are the same as in (A) and (B). [file peerj-11-16351-s001.png]
